# In-situ remediation of nitrogen and phosphorus of beverage industry by potential strains *Bacillus* sp. (BK1) and *Aspergillus* sp. (BK2)

**DOI:** 10.1038/s41598-021-91539-y

**Published:** 2021-06-10

**Authors:** Anne Bhambri, Santosh Kumar Karn, R. K. Singh

**Affiliations:** 1 Department of Biochemistry and Biotechnology, Sardar Bhagwan Singh University (Formerly, Sardar Bhagwan Singh Post Graduate Institute of Biomedical Science and Research), Balawala, Dehradun, 248161 India; 2 Department of Biotechnology, Shri Guru Ram Rai University, Patel Nagar, Dehradun, Uttarakhand 248001 India

**Keywords:** Applied microbiology, Biofilms, Industrial microbiology

## Abstract

The bioremediation of beverage (treated and untreated) effluent was investigated in the current study by using the potential strains of *Bacillus* sp. (BK1) and *Aspergillus* sp. (BK2). Effluent was collected from the beverage industry (initial concentration of nitrogen were 3200 ± 0.5 mg/L and 4400 ± 0.6 mg/L whereas phosphorus were 4400 ± 2 mg/L and 2600 ± 1 mg/L in treated and untreated effluent correspondingly). Further, the BK1 and BK2 exhibited high removal competence after 1 week of incubation; BK1 removed phosphorus 99.95 ± 0.7% and BK2 95.69 ± 1% in treated effluent while nitrogen removed about 99.90 ± 0.4% by BK1 and 81.25 ± 0.8% by BK2 (initial concentration of phosphorus 4400 ± 2 mg/L and nitrogen 3200 ± 0.5 mg/L). Next, in the untreated effluent BK1 removed 99.81 ± 1% and BK2 99.85 ± 0.8% of phosphorus while removed nitrogen 99.93 ± 0.5% by BK1 and 99.95 ± 1.2% by BK2 correspondingly, (initial concentration of phosphorus 2600 ± 1 mg/L and nitrogen 4400 ± 0.6 mg/L). The physiochemical composition of sample such as pH, total carbohydrates, total proteins, total solids of treated and untreated effluent were also analysed before and after treatment of both the samples. BK1 and BK2 increased the pH by 8.94 ± 0.3 and 9.5 ± 0.4 correspondingly in treated effluent whereas 6.34 ± 0.5 and 7.5 ± 0.2 correspondingly in untreated effluent (initial pH of treated and untreated effluent 7.07 ± 0.8 and 4.85 ± 0.3 correspondingly). Total Carbohydrates removed about 17,440 ± 4.6 mg/L and 10,680 ± 3.2 mg/L by BK1 and BK2 correspondingly in treated effluent whereas 18,050 ± 3.5 mg/L and 18,340 ± 2.3 mg/L correspondingly in untreated effluent (initial concentration of treated and untreated effluent 25,780 ± 1.6 mg/L and 35,000 ± 1.5 mg/L correspondingly) while BK1 and BK2 removed total proteins by 30.336 ± 4.6 mg/L and 40.417 ± 2.3 mg/L correspondingly in treated effluent whereas 18.929 ± 1.2 mg/L and 17.526 ± 0.8 mg/L correspondingly in untreated effluent (initial concentration of treated and untreated effluent 49.225 ± 1.5 mg/L and 20.565 ± 1 mg/L correspondingly). Next, total solids removed by BK1 and BK2 2.5 ± 0.3 mg/L and 1.6 ± 0.6 mg/L correspondingly in treated effluent whereas 5.5 ± 0.8 mg/L and 4.6 ± 0.6 mg/L in untreated effluent (initial concentration of treated and untreated effluent 5.6 ± 1.5 mg/L and 9.48 ± 1.2 mg/L correspondingly). Both the strains BK1 and BK2 are highly efficient in the nitrogen and phosphorus removal therefore this strain may be applied for the potential remediation.

## Introduction

A lot of attention has been given in developing countries for nutrients such as nitrogen and phosphorus for their increasing discharge from industrial, municipal, agricultural, household wastewater and anthropogenic activities^1, 2^. All over the world, nitrogen cycle has some significant changes in last 200 years. Over the large areas of earth, there is now elevated availability and mobility of nitrogen. Additionally, inorganic nitrogen like nitrates invade into the environment mainly in the aquatic systems via various anthropogenic activities like inappropriate treatment of industrial wastes, urban and agricultural runoff, leaching of waste in surface water, effluents of sewage, pesticides usage, organic or inorganic compounds^3, 4^. All over the world, if the concentration of nitrate elevated from the acceptable limit as mentioned in international standard is a major environmental concern in various sources of water. The one of the major sources of nitrate pollution is also the extensive use of fertilizers in agriculture. Ions of nitrate are highly soluble in water and cannot bind well with the soil particles due to which nitrate ions can be easily enter into the surface water and ground water^5, 6^. Due to excess concentration of nitrate in water causes lots of serious environmental problems such as lake eutrophication, red tides and also to human health such as stomach cancer, infant mortality, birth defects whereas ions of nitrates also cause methemoglobinemia or blue-baby disorder which are the major health problems in human beings mainly in infants and pregnant women and this happens due to the reduction of oxygen-carrying limit of blood^7–10^.

Concentration of nitrate is an important index for both wastewater as well as drinking water. According to world health organisation (WHO), the tolerance limit of nitrate content in drinking water is 40 mg/L and the phosphate content is < 0.5 mg/L^9, 11^. According to U.S. Environmental Protection Agency (USEPA) and WHO maximum contaminant level goal in drinking water have set in many countries i.e., 10 mg/L^12, 13^. In nature, phosphate and nitrate are the usual form of phosphorus and nitrogen correspondingly and in order to control the eutrophication, it considered as the principal nutrients that needs to be eliminated^14^. Inorganic nitrate can be removed by using most common procedure called denitrification, which leads to the reduction of nitrate to nitrogen by using bacteria and fungi where as the most common procedure for the removal of inorganic phosphate is physiochemical dephosphatization.

**Figure 1:**
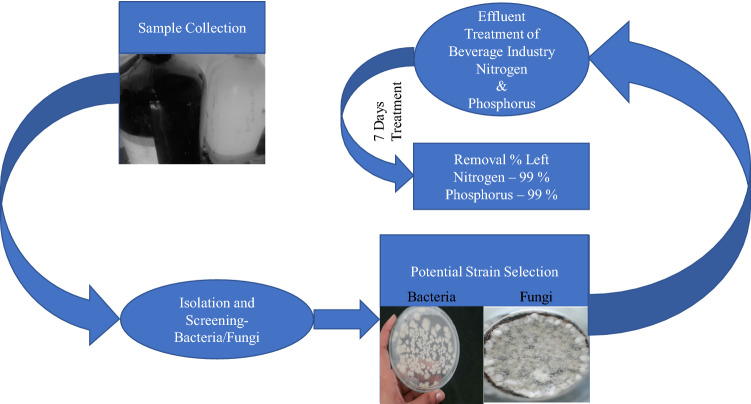
Flow chart of the current work.

There are several physical and chemical methods that are used for the removal of nitrate ions and phosphate from the wastewater, these are chemical precipitation^15^, reverse osmosis^16^, catalytic reduction^17^, adsorption method^18^, electrocoagulation^19^, membrane process^20^, ion-exchange^21^ etc. Among all these methods, Ion-exchange (IX) is considered to be a feasible process using anion exchange resin for its fast kinetics and high selectivity^22, 23^. IX methods have been suggested by Environmental Protection Agency (EPA) for the removal of nitrates from the groundwater and these problems have limited their industrial applications^24^. However, due to significant consumption of chemicals and energy, high operational cost of physiochemical process is avoided these types of removal methods cause less interest in these common processes^25, 26^. Biological method is also one of the important and widely used method for the removal of nitrogen and phosphorus from the wastewater before it discharged into the field, ground or surface water^27, 28^. It is economically attractive, simple and well accepted method that used for the biodegradation of organic contaminants accepted by the public. There are large number of species used in the mixed cultures called consortiums or pure cultures i.e., bacteria *Azospira* sp.^29^, *Acinetobacter* sp.^30^,* Vibrio* sp.^31^, *Bacillus cereus*, *Bacillus amyloliquefaciens* and *Pseudomonas stutzeri*^32^ and fungi such as *White-rot fungus, Aspergillus luchuensis*^33^, *Trametes versicolor, Pleurotus ostreatus* and *Trichoderma harzianum*^34^. Above mentioned fungi produce a wide variety of extracellular enzymes having high capacity of biodegradability and eliminate efficiently the biodegradable organic matter such as NH_3_, NH_4_^+^ etc^35^. Therefore, our main objective was to use the potential indigenous strains for the removal of nitrogen and phosphorus from the industrial effluents and from the polluted sites in the nature. The outline of the current work is mentioned in figure 1.

## Materials and methods

### Sample preparation

Two different effluents were employed in the nutrients removal study from the beverage industry. This beverage (treated and untreated) effluent was collected from the Doon Valley Distillers (a beverage industry) in a pre-cleaned, sterilized polyethylene bottles located at Kuanwala, Dehradun (Longitude, 78.056°E and Latitude, 30.3148°N), Uttarakhand, India. Before the collection of samples, the bottles were cleaned with detergent solution and then washed with deionized water and dried it well in the air. The collected effluent was labelled properly, taken to the laboratory and stored at 4 °C prior to use. All the chemicals that used were purchased from RANKEM and HIMEDIA Mumbai, India and reagents were prepared by using double distilled water.

### Removal of nitrogen and phosphorus by potential strain

BK1 and BK2 were previously isolated and characterized as *Bacillus* sp. and *Aspergillus* sp. respectively, in the laboratory which is used for this study. BK1 was repeatedly sub-cultured before storage. During subculture medium was supplemented with nitrogen and phosphorus as a constituents into the medium such as NaNo_2_ and Na_2_HPO_4_. Next, bacterial culture was stored by deep-freezing while the culture was stocked in broth medium with glycerol. Glycerol was added to prevent the desiccation of the bacterial cells. Similarly, BK2 was also repeatedly sub-cultured and maintained on potato dextrose agar (PDA) medium.

### Physiochemical properties of soil sample

The physiochemical analysis of soil sample was performed by various techniques from which BK1 and BK2 were isolated. The evaluation was focused on following physical and chemical parameters such as colour of the soil, texture, conductivity, porosity, total dissolved solids (TDS) and pH, chlorine, magnesium, organic matter, sodium, total alkalinity, calcium, potassium, nitrate-nitrogen, phosphate-phosphorus, carbon and hydrogen respectively. The analysis of the above-mentioned parameters was followed by American Public Health Association (APHA) manual^36^.

### Physiochemical characterization of effluent

The physiochemical analysis of the beverage effluent (treated and untreated) sample was performed by various techniques that have been carried out as per standard procedure prescribed by APHA manual^36^. The evaluation was focused on the following parameters and their concentrations were measured in each sample such as nitrogen, phosphorus, total carbohydrates, total proteins were determined by Anthrone test, Bradford method and total solids were measured by using UV–VIS Spectrophotometer (Systronics 119), India and pH were determined by using portable instrument pH510, EUTECH instrument, (Cyber Scan, Malaysia). Nitrogen, phosphorus determination protocol is mentioned below.

### Inoculum preparation

For culture preparation, nutrient broth was prepared, autoclaved and then culture was inoculated from freshly sub cultured plates into the liquid medium containing 50 ml nutrient broth. After inoculation, incubated for overnight incubation at 37 °C. Further, after incubation the growth was measured by UV–VIS Spectrophotometer (Systronics119), India at 600 nm in each flask. Next, for fungi, growth was observed visually and 2 mm of disc was used for inoculums for the removal experiment.

### In-situ remediation of nitrogen and phosphorus

#### Treatment by *Bacillus* sp. (BK1)

To treat the effluent by BK1, 100 ml of treated effluent were taken in four different flasks and 10% of broth culture (Inoculum) was added in each flask. Simultaneously, 100 ml of untreated effluent were also taken in four different flasks and 10% inoculums included in each flask. Further, all the flasks were kept on rotary shaker for at least 1 week. Next, to count the colonies, nutrient agar was again prepared, autoclaved and then poured it in a pre-autoclaved plate, solidified them and then serially diluted. Further, incubated overnight at 37 °C to observe the growth.

#### Treatment by *Aspergillus* sp. (BK2)

To treat the effluent by *Aspergillus* sp., fungal culture was excise a disc of about 2 to 5 mm size (fungi) and inoculated in the untreated effluent and treated then incubated the flasks for at least 1 week under static conditions and temperatures at 28 °C. Further, characterized all the parameters mentioned above. These cultures were used for the nitrogen and phosphorus removal from treated and untreated effluent. Only one culture was selected for the further study.

### Analytical techniques

Nitrogen and phosphorus recovery from the treated and untreated sample was determined by the following techniques.

### Phosphorus test

Sample solution was made neutral with 0.5 N NaOH and 2.0 N CH_3_COOH. Prepared different concentrations of standard PO_4_^3−^ say 0.1 ml to 1.0 ml in 100 ml volumetric flasks. Further, taken appropriate amount of sample in 100 ml of volumetric flask. Added 10.0 ml molybdate solution (2.5 g of A.R. Na_2_MO_4_2H_2_O in 100 ml of 10 N sulphuric acid) in each volumetric flask and 4.0 ml of hydrazine sulphate reagent (0.375 g of A.R. hydrazine sulphate in 250 ml of distilled water) in it. Adjusted the volume up to 100 ml with distilled water and mixed well. Boiled the contents in water bath for 10 min. After cooling the volumetric flask, rapidly shake and mixed well. Measured the absorbance at 830 µm against deionised water. Constructed the calibration curve, using the standard phosphate solution^36^.

### Calculation

Calculate the PO_4_^3−^ content of the sample

$$\mathcal{P}{\mathcal{O}}_{4}^{-3 } \text{mg}/\text{L} = \frac{\mathrm{\rm X} (\text{mg})}{\Upsilon (\text{ml})} \times \frac{100 \times {10}^{-3 }\times 100 \%}{\mathcal{Z}(\text{g})}$$

where, X is concentration in mg corresponding to Y ml of sample solution (100 ml of total sample volume) and Z is amount of sample in gm dissolved in 100 ml.

### Nitrogen test

Taken accurately 1 ml of effluentinto the kjeldal flask. The quantity of the sample taken such that ammonia liberated neutralized not more than 40 ml of standard sulphuric acid and had taken in the flask in which tube dips. Added 5.0 g of potassium sulphate or anhydrous sodium sulphate, 68.0 mg of the selenium powder, 168 mg of copper sulphate (CuSO_4_·5H_2_O) in it. Added 15.0 ml of conc. H_2_SO_4_ acid. Placed the kjeldal flask in an inclined position and closed the flask with a loose fitted to prevent the loss of sulphuric acid or entry of dust. Heated the mixture into the fumed cupboard until the initial froth had ceased. Heated the mixture to the boiling point. Continued the boiling freely until the solution becomes clear and then boiled for a further period of about 2 h. Cooled the contents of the flask at room temperature. Assembled the distillation assembly for nitrogen estimation. Boiled the distilled water in the flask of one litre capacity continuously so that once steam generated not give back pressure. Added 25.0 ml of 0.01 N HCl, 2.3 drops of methyl red indicator in the conical flask (100 ml capacity) in which tube connected to lower end of the condenser was dipped. Further, transferred the whole digested mixture through the funnel. Rinsed the kjeldal flask and funnel with distilled water and then added 33% NaOH (20 ml) solution. Tightened the screw near the funnel. Further, heated the water continuously for 25–30 min. so that level of 0.01 N HCl get almost doubled in the flask so that the steam generated collected in 0.01 N HCl flask. Further, removed the condenser lid from the liquid portion of the flask (above the surface of 0.01 N HCl) and stopped boiling of water so that steam generated creates a back pressure. The whole contents come in the vacuum jacket flask from digestion flask by back pressure. Discarded the liquid by opening the screw from vacuum jacket flask. Further, titrated the 0.01 N HCl flasks with already standardized 0.01 N NaOH by added a few drops of indicator^36^.

### Calculation

$$Nitrogen \; percent \; by \; mass = \frac{1.4 \left({V}_{2 }- {V}_{1}\right) N}{W}$$

where,V_1_ = Volume in ml of standard NaOH soln. used to neutralize the excess acid in the determination with the sample, V_2_= Volume in ml of standard NaOH soln. used to neutralize the excess acid in blank determination, N=Normality of standard NaOH solution, W = Mass in gram of the sample taken for the test.

### Atomic absorption spectroscopy

By using flame atomic absorption spectrometer, analysis of nitrogen and phosphorus was performed. In 5 ml of concentrated HNO_3,_ 100 ml of samples were digested on a hot plate in a fume hood. Until the digestion completed, samples were boiled gently to the lowest possible volume, which further shown by a light-coloured, clear solution. In a volumetric flask, the coolest sample was made up to a volume of 100 ml after the complete digestion. Further, sample were filtered by using Whatman™ qualitative 1 filter paper (125 mm diameter * 100 circles) and analysed in a flame atomic absorption spectrophotometer for the nitrogen and phosphorus contents. For the preparation of calibration standards by dilution, 1000 mg/L of stock metal standard solutions were used during analysis. The mean values of three consecutive replicates were adopted for each measurement. Next, samples were diluted using deionized water in case of high concentrations.

### Data analysis

All experiments were performed in three replicates and statistical analysis was performed using GraphPad Prism (version 4.03) software (GraphPad, CA, USA).

## Result

### Microorganisms, physiochemical characterization of soil sample

*Bacillus* sp. (BK1) and *Aspergillus* sp. (BK2) was enriched with nitrogen and phosphorus and used in the current study further, repeatedly sub-cultured and maintained in glycerol stock solution. Soil sample was physiochemically characterized both by physical and chemical parameters. In physical parameters, we observed the colour of the soil sample as greyish-brown and the texture of soil was loamy soil. Further, conductivity of the soil sample was found to be 1.02 ± 0.5 mg/kg and the porosity was 0.178 ± 0.23%. Next, we determined the total dissolved solids and was found to be 1227.9 ± 1.2 mg/kg whereas in chemical parameters we observed the pH of the soil sample by electrometric method and was found to be 7.8 ± 0.5. Further, we determined magnesium and was found to be 435.2 ± 2.6 mg/kg. Next, organic matter was determined and was found to be 39.6 ± 1.5 mg/kg whereas sodium was 224 ± 2.1 mg/kg. Further, we determined the total alkalinity and were found to be 1390 ± 0.9 mg/kg while calcium was observed 526.8 ± 3.4 mg/kg. Next, potassium was determined and found to be 126.3 ± 1.2 mg/kg whereas nitrate-nitrogen and phosphate-phosphorus was found to be 18 ± 0.4 and 1.7 ± 0.2 mg/kg respectively. Further details mentioned in Table 1.

**Table 1:** Physiochemical properties of soil.

Physiochemical properties of soil		
Physical parameters		
S. no.	Parameters	Values (mg/kg)
1	Colour	Greyish-brown
2	Texture	Loamy soil
3	Conductivity (µmho)	1.02 ± 0.5
4	Porosity (%)	0.178 ± 0.23
5	Total dissolved solids (TDS)	1227.9 ± 1.2

Chemical parameters		
S. no.	Parameters	Values
1	pH	7.8 ± 0.5
2	Magnesium (Mg^2+^)	435.2 ± 2.6
3	Organic matter (%)	39.6 ± 1.5
4	Sodium (Na^+^)	224 ± 2.1
5	Total alkalinity (TA)	1390 ± 0.9
6	Calcium (Ca^2+^)	526.8 ± 3.4
7	Potassium (K^+^)	126.3 ± 1.2
8	Nitrate-nitrogen (NO_3_^−^)	18 ± 0.4
9	Phosphate-phosphorus (PO_4_–P)	1.7 ± 0.2

### Physiochemical characterization of effluent

Beverage effluents both treated and untreated sample was physiochemically characterized. pH of the beverage effluent was determined by electrometric method and found that before treatment the pH of treated and untreated effluent was 7.07 ± 0.8 and 4.85 ± 0.3 correspondingly, whereas after treatment by BK1 and BK2, the overall pH of the effluent increased both in treated and untreated beverage effluent. More specifically after treatment by BK1, the pH of treated and untreated effluent was 8.94 ± 0.3 and 6.34 ± 0.5 respectively, whereas, after treatment by BK2, pH of treated and untreated effluent was 9.5 ± 0.4 and 7.5 ± 0.2 correspondingly.

Additionally, we determine total carbohydrate from the beverage effluent by using anthrone test and found that before treatment the removal activity from the treated and untreated effluent were 25,780 ± 1.6 and 35,000 ± 1.5 mg/L correspondingly. However, we found that the level of carbohydrate reduced both in treated and untreated effluent after the treatment by BK1 and BK2. From the treated and untreated effluent, the removal of total carbohydrates after the treatment by BK1 were 17,440 ± 4.6 and 18,050 ± 3.5 mg/L, respectively, whereas the removal of total carbohydrates after the treatment by BK2 from the treated and untreated effluent were 10,680 ± 3.2 and 18,340 ± 2.3 mg/L, correspondingly. Next, we determined the total protein removal activity by Bradford method and found the content of protein both in treated and untreated effluent before the treatment were 49.225 ± 1.5 and 20.565 ± 1 mg/L, correspondingly. Later, after BK1 and BK2 treatment, we observed that the level of total protein reduced in both treated and untreated effluent. After the treatment by BK1, the removal of total protein from the treated and untreated effluent were 30.336 ± 4.6 and 18.929 ± 1.2 mg/L, respectively, whereas, after BK2 treatment it were 40.417 ± 2.3 and 17.526 ± 0.8 mg/L, correspondingly.

From both the treated and untreated effluent, total solids were also observed and found that both in treated and untreated beverage effluent the amount of total solids before the treatment were 5.6 ± 1.5 and 9.48 ± 1.2 mg/L, correspondingly, but after the treatment by BK1 & BK2, the total amount of solids were found to be reduced both in treated and untreated effluent 2.5 ± 0.3 and 5.5 ± 0.8 mg/L, correspondingly, by BK1 and 1.6 ± 0.6 and 4.6 ± 0.6 mg/L, correspondingly, by BK2 due to the production of biomass. Details mentioned in Tables 2 and 3.

**Table 2:** Physiochemical characterization of beverage effluent.

S. no.	Effluents		PH	Total proteins (mg/L)	Total carbohydrates (mg/L)	Total solids (mg/L)	Nitrogen (mg/L)	Phosphorus (mg/L)
1	Untreated effluent	Before treatment	4.85 ± 0.3	20.565 ± 1	35,000 ± 1.5	9.48 ± 1.2	4400 ± 0.6	2600 ± 1
2	Treated effluent	Before treatment	7.07 ± 0.8	49.225 ± 1.5	25,780 ± 1.6	5.6 ± 1.5	3200 ± 0.5	4400 ± 2

**Table 3:** Characterization of effluent after removal by BK1 and BK2.

S. no.	Effluents		PH	Total proteins (mg/L)	Total carbohydrates (mg/L)	Total solids (mg/L)
1	Untreated effluent	Treatment by BK1	6.34 ± 0.5	18.929 ± 1.2	18,050 ± 3.5	5.5 ± 0.8
		Treatment by BK2	7.5 ± 0.2	17.526 ± 0.8	18,340 ± 2.3	4.6 ± 0.6
2	Treated effluent	Treatment by BK1	8.94 ± 0.3	30.336 ± 4.6	17,440 ± 4.6	2.5 ± 0.3
		Treatment by BK2	9.5 ± 0.4	40.417 ± 2.3	10,680 ± 3.2	1.6 ± 0.6

### Nitrogen and phosphorus removal by BK1

**Figure 2:**
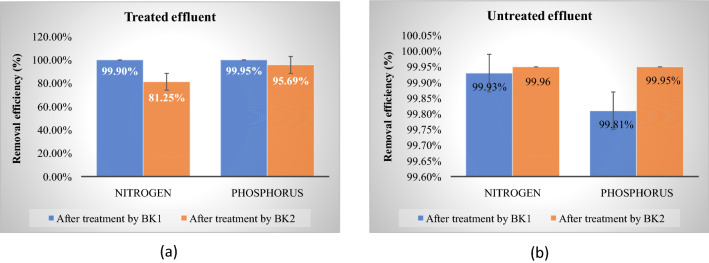
Removal of nitrogen and phosphorus (%) by BK1 and BK2.

Phosphorus removal activity was quantified from the effluent of beverage and found that the phosphorus content before treatment from the treated and untreated effluent were 4400 ± 2 and 2600 ± 1 mg/L, correspondingly, whereas after the treatment by BK1, we found that the level of phosphorus decreased both in the treated and untreated effluent by 99.95 ± 0.7% and 99.81 ± 1%, respectively. Further, nitrogen by BK1 was estimated from the beverage effluent and found that before treatment the content of nitrogen from the treated and untreated effluent were 3200 ± 0.5 mg/L and 4400 ± 0.6 mg/L, correspondingly, whereas, after the treatment by BK1, we observed that the level of nitrogen decreased both in treated and untreated effluent and found to be 99.90 ± 0.4% and 99.93 ± 0.5% correspondingly. Details of removal are mentioned in Table 4 and Fig. 2.

**Table 4:** Removal of nitrogen and phosphorus by strains BK1 and BK2.

S. no.	Effluents		Phosphorus (mg/L)	Phosphorus removal in (%)	Nitrogen (mg/L)	Nitrogen removal in (%)
1	Untreated effluent	Before treatment	2600 ± 1	–	4400 ± 0.6	**–**
		Treatment by BK1	5 ± 0.8	99.81 ± 1	3 ± 0.2	99.93 ± 0.5
		Treatment by BK2	4 ± 0.6	99.85 ± 0.8	2 ± 1	99.95 ± 1.2
2	Treated effluent	Before treatment	4400 ± 2	**–**	3200 ± 0.5	
		Treatment by BK1	2 ± 0.5	99.95 ± 0.7	3 ± 0.2	99.90 ± 0.4
		Treatment by BK2	190 ± 0.3	95.69 ± 1	600 ± 0.5	81.25 ± 0.8

### Nitrogen and phosphorus removal by BK2

Further determined the removal of phosphorus by BK2 and found that after the treatment by BK2 the level of phosphorus reduced both in treated and untreated effluent and were found to be 95.69 ± 1% and 99.85 ± 0.8% correspondingly. Next, we determined the removal of nitrogen and found that after the treatment by BK2, the level of nitrogen decreased both in treated and untreated effluent by 81.25 ± 0.8% and 99.95 ± 1.2% correspondingly. Details mentioned in Table 4 and Fig. 2.

### Colony forming unit at different time intervals

We observed the colony forming unit (cfu) of bacterial cells at different time intervals and found that at 12 h time interval, the cfu of treated and untreated effluent were 0.8 × 10^2^ and 0.5 × 10^2^ cfu/ml respectively whereas at 24 h time interval, the cfu of treated and untreated effluent were 2.8 × 10^2^ and 2.3 × 10^2^ cfu/ml respectively. Further, at 48 h time interval, the cfu of treated and untreated effluent were 2.7 × 10^3^ and 3.2 × 10^3^ cfu/ml respectively while at 72 h time interval, the cfu of treated and untreated effluent were 4.6 × 10^4^ and 3.8 × 10^4^ cfu/ml respectively.

Next, at 96 h time interval, we observed that the cfu of treated and untreated effluent were 6.8 × 10^5^ and 5.4 × 10^5^ cfu/ml respectively while at 120 h, the cfu of treated and untreated effluent were 7.4 × 10^6^ and 6.8 × 10^6^ cfu/ml respectively. Further, details mentioned in Table 5.

**Table 5:** Colony forming unit observed in (cfu/mL) at different time intervals.

S. no.	Time interval	Treated effluent (cfu/mL)	Untreated effluent (cfu/mL)
1	12 h	0.8 × 10^2^	0.5 × 10^2^
2	24 h	2.8 × 10^2^	2.3 × 10^2^
3	48 h	2.7 × 10^3^	3.2 × 10^3^
4	72 h	4.6 × 10^4^	3.8 × 10^4^
5	96 h	6.8 × 10^5^	5.4 × 10^5^
6	120 h	7.4 × 10^6^	6.8 × 10^6^

### Nitrogen and phosphorus removal by BK1 and BK2 at different time interval

We observed the removal rate of nitrogen and phosphorus by BK1 and BK2 at different time intervals. BK1 removed nitrogen 2210 ± 6.5 mg/L and BK2 2183 ± 3.9 mg/L in treated effluent while phosphorus removed about 2602 ± 10.3 mg/L by BK1 and 3121 ± 8.4 mg/L by BK2. Next, in untreated effluent BK1 removed 2511 ± 5.9 mg/L and BK2 2698 ± 7.4 mg/L of nitrogen and phosphorus 2081 ± 9.5 mg/L by BK1 and 1925 ± 6.8 mg/L by BK2 correspondingly at 24 h time interval.

Further, at 72 h time interval, BK1 removed nitrogen 1011 ± 4.3 mg/L and BK2 1180 ± 4.2 mg/L in treated effluent whereas phosphorus removed about 822 ± 6 mg/L by BK1 and 1640 ± 8.3 mg/L by BK2. Next, in untreated effluents BK1 removed 623 ± 8 mg/L and BK2 853 ± 5 mg/L of nitrogen and phosphorus 500 ± 8.6 mg/L by BK1 and BK2 925 ± 7.3 mg/L correspondingly.

At 120 h time interval, BK1 removed nitrogen 6 ± 2 mg/L and BK2 610 ± 10 mg/L in treated effluent while phosphorus removed about 5 ± 3 mg/L by BK1 and 200 ± 10 mg/L by BK2. Next, in untreated effluents BK1 removed 5 ± 2 mg/L and BK2 5 ± 3 mg/L of nitrogen and phosphorus 10 ± 5 mg/L by BK1 and BK2 7 ± 3 mg/L correspondingly. Further details mentioned in Table 6.

**Table 6:** Removal rate of nitrogen and phosphorus at different time intervals.

S. no.	Effluents		Phosphorus (mg/L)						Nitrogen (mg/L)					
			12 h	24 h	48 h	72 h	96 h	120 h	12 h	24 h	48 h	72 h	96 h	120 h
1	Untreated effluent	Treatment by BK1	2450 ± 10	2081 ± 9.5	1150 ± 7.9	500 ± 8.6	180 ± 6.2	10 ± 5	3802 ± 4.7	2511 ± 5.9	1526 ± 11	623 ± 8	120 ± 6.8	5 ± 2
		Treatment by BK2	2415 ± 8.2	1925 ± 6.8	1432 ± 9.1	925 ± 7.3	312 ± 5.8	7 ± 3	3795 ± 6.8	2698 ± 7.4	1753 ± 9.4	853 ± 5	352 ± 6.2	5 ± 3
2	Treated effluent	Treatment by BK1	3500 ± 9.4	2602 ± 10.3	1715 ± 7.2	822 ± 6	132 ± 7.1	5 ± 3	2816 ± 9.2	2210 ± 6.5	1628 ± 8	1011 ± 4.3	405 ± 9	6 ± 2
		Treatment by BK2	3826 ± 10.7	3121 ± 8.4	2238 ± 6.5	1640 ± 8.3	740 ± 5.4	200 ± 10	2915 ± 7.6	2183 ± 3.9	1784 ± 6.5	1180 ± 4.2	980 ± 5.5	610 ± 10

## Discussion

### Physiochemical characterization of soil sample

The physiochemical characterization of soil sample was investigated and found 18 ± 0.4 mg/kg of nitrate-nitrogen and 1.7 ± 0.2 mg/kg of phosphate-phosphorus in the agricultural soil at pH 7.8 ± 0.5. The texture of the soil sample was loamy soil and having organic matter of about 39.6 ± 1.5% while potassium 126.3 ± 1.2 mg/kg. Previously Pokhriya et al. observed 1000 ± 0.07 mg/kg of total nitrogen and 7.2 ± 0.05 mg/kg of phosphorus in the effluent affected agricultural soil at pH 8.1 ± 0.7. The texture of the soil was loam and having organic matter of about 8500 ± 0.05 mg/kg while potassium 91.2 ± 1.03 mg/kg^37^ whereas, Bilal and Reyaz investigated 19.5 mg/kg of nitrate-nitrogen and 0.50 mg/l of phosphate-phosphorus in the agricultural soil at pH 8. The texture of the soil was slity—clay and organic matter of about 5.32% while potassium 150 mg/kg^38^.

### Physiochemical characterization of effluent

There is a general interest in studying the diversity of bacteria and fungi that are capable of degrading the pollutants which have varied effects on the environment. Efforts have been made for the characterization of microbial and fungal communities and their responses to the pollutants for the isolation of possible degrade^39^. By having specific degradation capabilities, bacteria and fungi can be isolated by adding the compound of interest for the enrichment of cells taken from the beverage effluent sample. Further, isolates can be then obtained on the culture media. In this way, one may select the bacteria and fungi that can survive in the presence of pollutant compounds. The present study shows the removal of biological nutrients from the beverage effluent by *Bacillus* sp. (BK1) and *Aspergillus* sp. (BK2). In the current study, two potential strains BK1 and BK2 are selected for further study. The effluents are physiochemically characterized and has been shown in result section of Table 1. Potentially nitrogen and phosphorus is remediated from the sample effluent.

### Treatment of nitrogen and phosphorus

The removal of nitrogen and phosphorus from the wastewater has become an emergent concern globally because the elevated amount of these compounds causes eutrophication in natural water. An activated sludge process and many other processes are used for the removal of nitrogen and phosphorus from the effluents but it is often the case that the effluent from the wastewater treatment plants has remaining phosphorus and nitrogen in the form of ammonium and nitrate. Therefore, a post-treatment process also requires for the removal of nitrogen and phosphorus from the effluents^40, 41^. In the present study, the removal efficiency of nitrogen and phosphorus were monitored by BK1 and BK2 from the treated and untreated effluent sample of beverage industry and found removal in both the effluent by both the strain above 90%. Excessive release of nitrogen and phosphorus from human activities into runoff which imposes great risk to the ecosystem and degrades the fresh water quality^42–44^. Previously different scientist tried to find the efficient strain for the removal of phosphorus and nitrogen like, Li et al. investigated that microorganisms i.e., *Candida tusaccumulibacter, Zoogloea* and *Dechloromonas* removal efficiency of PO_4_^3–^–P reached up to 93.64% at an initial concentration of 0.7 mg/L and 92.34% of NO_3_^−^–N at an initial concentration of 0.6 mg/L correspondingly^45^. Ge et al. investigated that the removal efficiency of total nitrogen 69.4 ± 21.4% at an initial concentration of 4.0 ± 3.2 mg/L whereas removed total phosphorus 87.7 ± 14.2% at an initial concentration of 0.25 ± 0.20 mg/L correspondingly by using the bacteria *Anaeromyxobacter* (4.9%), *Ramlibacter* (4.8%), *Defluviicoccus* (4.2%), *Azoarcus* (3.7%) and *Geobacter* (3.4%)^46^.

Nitrogen and phosphorus are a major problem in many industries such as milk whey processing wastewater, meat processing wastewater, domestic wastewater and real municipal wastewater etc. Therefore, several efforts have been made for the removal of nitrogen and phosphorus from the various industries. Ye et al. found that the phosphorus accumulating fungi *Mucor circinelloides* have maximum utilization of 40.1% for phosphorus from the waste streams^47^ whereas Dalecka et al. found the removal efficiency of phosphorus 99.9% by *Trametes versicolor* after 6-h of incubation period whereas 99.9% of phosphorus removed by *Aspergillus luchuensis* after an incubation of 24 h at an initial concentration of 2600 mg/L whereas they found that the concentration of nitrogen increases from 250 to 2300 mg/L by *Trametes versicolor* and 200 mg/L to 1400 mg/L by *Aspergillus luchuensis* correspondingly from non-sterile municipal wastewater^33^. Posadas et al. found that 70 ± 8% of nitrogen and 85 ± 9% of phosphorus were removed in the algal–bacterial bioreactor while the treatment of domestic wastewater at 10 day of hydraulic retention time and also observed that no phosphorus were removed while treated with bacterial biofilm^48^ whereas Kim et al. found that the removal efficiency of total nitrogen (89.3%) and total phosphorus (94.9%) at the optimal condition in summer whereas 84% of total nitrogen and 88.3% of total phosphorus removed in winter at an initial concentration of 7.1 ± 0.5 mg/L of total nitrogen and 1.1 ± 0.1 mg/L of total phosphorus correspondingly in marine wastewater with a pilot plant-scale sequencing batch reactor system^49^.

Above result suggest that the average value of nitrogen and phosphorus removal efficiency in 1-week period of working and maintenance from the beverage effluent sample (treated and untreated) found that the removal efficiency of nitrogen that reached up to 99.90 ± 0.4% after the treatment by BK1 and 81.25 ± 0.8% after the treatment by BK2 in treated effluent at an initial concentration of 3200 ± 0.5 mg/L whereas in untreated effluent, the removal efficiency of nitrogen was reached up to 99.93 ± 0.5% after the treatment by BK1 and 99.95 ± 1.2% was removed after the treatment by BK2 at an initial concentration of 4400 ± 0.6 mg/L. Previously Chen et al. found that the co-culture of two strains fungus *Penicillium citrinum* WXP-2 and bacterium *Citrobacter freundii* WXP-9 that formed fungus-bacterium pellets promoted the removal efficiency of total nitrogen 81.73% at an initial concentration of 101.56 mg/L by enhancing the enzyme activity and electron transfer^50^.

Further, we observed the phosphorus removal efficiency that reached up to 99.95 ± 0.7% after the treatment by BK1 and 95.69 ± 1% after the treatment by BK2 in treated effluent at an initial concentration of 4400 ± 2 mg/L whereas the removal efficiency of phosphorus in untreated effluent was reached up to 99.81 ± 1% after the treatment by BK1 and 99.85 ± 0.8% after the treatment by BK2 at an initial concentration of 2600 ± 1 mg/L. According to Papadopoulos et al. determined the removal efficiency of nitrogen and phosphorus from the brewery wastewater by using cyanobacterial-bacterial aggregates and found that 70% of total phosphorus and 90% of ammonium were removed when grown under the condition of optimum pH and temperature (28 ± 1 °C)^51^ whereas He et al. investigated that the 83.9% of phosphorus and 46.5% of total nitrogen was removed by microbial treatment in 20-folds diluted dairy manure wastewater by fungal biomass in 12 h^52^**.** Bawiec demonstrated that the removal efficiency of total nitrogen was 82–83% and total phosphorus was 83–84% from the waste water treatment plants by hydroponic technology^53^, whereas Lee et al. investigated the effect of photoperiod in lab-scale photobioreactors having algal bacterial consortia for the reduction of organic nutrients from the municipal wastewater and found that after 12 days of batch operation 35–88% of nitrogen and 43–89% of phosphorus were removed from the influents^54^. According to Reza and Cuenca, on the basis of well-known bacterial species of genus *Nitrosomonas a*nd *Nitrobacter* that involved in the nitrification/denitrification for the biological nutrients removal processes and *Candida tusaccumulibacter* phosphates that responsible for the removal of biological phosphorus found that 90% of total nitrogen and total phosphorus removed at an initial concentration of 272 ± 7.5 mg/L and 32.6 ± 0.7 mg/L correspondingly^55^ whereas Wang et al. demonstrated the removal efficiency of total nitrogen (80%) by *Agrobacterium* sp. LAD9 at an initial concentration of 8.8 mg/L into a single biological aerated filter (BAF) for the bioaugmented treatment of municipal wastewater^28^. Hultberg and Bodin found that on 13^th^ day, the maximum removal of total nitrogen was 43% for *Trametes versicolor*, 52.5% for *Trichoderma harzianum* and 48.4% for *Pleurotusos treatus* whereas for phosphorus, the maximum reduction was found to be 28.3% for *Trametes versicolor* M_9912_, 39.5% for *Trichoderma harzianum* and 44% for *Pleurotusos treatus* from brewery wastewater^56^.

Liu et al. found that the anaerobic–aerobic-anoxic sequencing batch reactor system achieved the maximum removal efficiency of total nitrogen and total phosphorus i.e., 96.32% and 94.33% correspondingly by altering the cycle time of 6 h^57^ whereas Al-Dhabi et al. investigated that bacterial strain Al-Dhabi-17 characterized as *Stenotrophomonas maltophilia* Al-Dhabi-17 efficiency removed the nutrients from the wastewater than the other isolates and found that the removal efficiency of total nitrogen and total phosphorus were 88.4% and 97.9% respectively by the suspended growth sequencing batch reactor^58^. Sniffen et al. found that 9.18 mg/L of total nitrogen were removed per day in raw leachate by using the mixed culture of bacteria and algae^59^ whereas Zhang et al. observed that the phosphate-accumulating organism, *Arthrobacter* sp. HHEP_5_ removed 99% of phosphorus and 99.37% of nitrate isolated from the mariculture effluents^60^. Wang et al. demonstrated that the removal rate of total nitrogen and total phosphorus were 78.3% and 87.2% correspondingly after the treatment by *Chlorella-exiguobacterium* from the piggery wastewater treatment^61^.

Apart from the problem of nitrogen and phosphorus in industries, it also occurs in natural aquatic systems such as in lakes and reservoirs, the highest concentration of total nitrogen were found to be 1.65–10.81 mg/L in Shendinghe river followed by Sihe river that has an annual average content of 7.30 mg/L and is observed that the pollution of nitrogen in Sihe river and Shendinghe river are more serious as compared with that in other tributaries whereas the content of total phosphorus were found to be 0.03–0.56 mg/L followed by Shendinghe river that has an annual average content of 0.43 mg/L and it is observed that the contents of total phosphorus both in Sihe and Shendinghe river is higher than the grade III standard (0.2 mg/L), which determines that the pollution of phosphorus in Sihe and Shendinghe rivers is more serious than that in other tributaries^62^ which affects the lots of fish farming, turtles etc. That’s why we found the potential microbes and fungi that are much efficient for the removal of 99% of nitrogen and 99% of phosphorus then the previous ones. Therefore, it can be applied in many industries.

There are various mechanisms involved in the removal of nitrogen and phosphorus by bacteria and fungi. These are adsorption, biosorption, bioaccumulation, mineralization, ammonification, ammonia adsorption, nitrogen fixation, ammonia volatilization, nitrification, immobilization, ANAMMOX, nitrate-ammonification, organic nitrogen burial, denitrification, plant and microbial uptake, adsorption or precipitation, by biological storage in microorganisms, P, K and Fe mobilization via the production of siderophores and organic acids and Polyphosphate accumulation^63–67^. In nitrogen transformation, nitrification and denitrification are the two critical pathways. For nitrogen transformation and NH_3_ release, the fluctuation of ammonia–nitrogen in composts is an essential implication^68^. Ye et al. stated that the possible mechanism for the removal of phosphorus by fungi *Termetes versicolor* might be adsorption^47^. Dalecka et al. claimed that there might be the involvement of other mechanism for the removal of phosphorus i.e. cellular growth of fungi. They also stated that the mold *Aspergillus luchuensis* used biosorption mechanism for the removal of phosphorus while *Termetes versicolor* used both biosorption and metabolism mechanism^33^. Ahlgren et al. and Reitzel et al. stated that the mesotrophic lake Erken showed that reported for the phosphorus mineralization 69–71. Tong et al. cited that 90% of ammonium-nitrogen was removed due to anaerobic ammonia oxidant in anaerobic stage of hydrolysis-acidification tank^72, 73^. The exact mechanism for BK1 and BK2 is not known further studies are continued to know the detail mechanism. It is believed that BK1 and BK2 might also be using the biosorption, mineralization, ammonification, ammonia adsorption, nitrogen fixation, immobilization or nutrient utilization.

## Conclusion

Current work comes up with the highly efficient strains BK1 and BK2. BK1 removed 99.90 ± 0.4% of nitrogen and 99.95 ± 0.7% of phosphorus and BK2 removes 81.25 ± 0.8% of nitrogen and 95.69 ± 1% of phosphorus were removed from the treated effluent whereas 99.93 ± 0.5% of nitrogen and 99.81 ± 1% of phosphorus were removed by BK1 and 99.95 ± 1.2% of nitrogen and 99.85 ± 0.8% of phosphorus were removed by BK2 from the untreated beverage effluent within 7 days of treatment under the optimized conditions. After comparing results with the previous studies, it is found that current strain is more efficient than the previous report and further can be applied to many industrial effluents containing high nitrogen and phosphorus.

## References

[1] Bhatnagar A, Sillanpaa M (2011). A review of emerging adsorbents for nitrate removal from water. J. Chem. Eng. J..

[2] Li Q, Huang B, Chen X, Shi Y (2015). Cost-effective bio-regeneration of nitrate-laden ion exchange brine through deliberate bicarbonate incorporation. Water Res..

[3] Khatri N, Tyagi S (2015). Influences of natural and anthropogenic factors on surface and groundwater quality in rural and urban areas. Front. Life Sci..

[4] Baojing G, Ying G, Chang SX, Weidong L, Jie C (2013). Nitrate in groundwater of China: Sources and driving sources. Glob. Environ. Change..

[5] Bhatnagar A, Chinnasamy S, Singh M, Das KC (2010). Renewable biomass production by mixotrophic algae in the presence of various carbon sources and wastewater. Appl. Energy.

[6] Lin L, Zhang Y, Beckman M, Cao W, Ouyang T, Wang S, Li YY (2019). Process optimization of anammox–driven hydroxyapatite crystallization for simultaneous nitrogen removal and phosphorus recovery. Bioresour. Technol..

[7] Mclsaac GF, David MB, Gertner GZ, Goolsby DA (2011). Eutrophication: Nitrate flux in the Mississippi river. Nature.

[8] Greer FR, Shannon M (2005). Infant methemoglobinemia: The role of dietary nitrate in food and water. Pediatrics.

[9] Jiang H, Chen P, Luo S, Tu X, Cao Q, Shu M (2013). Synthesis of novel nanocomposite Fe_2_O_4_/ZrO_2_/chitosan and its application for removal of nitrate and phosphate. Appl. Surf. Sci..

[10] Abdel-Aziz MH, El-Ashtoukhy ESZ, Zoromba MS, Bassyouni M, Sedahmed GH (2019). Removal of nitrates from water by electrocoagulation using a cell with horizontally oriented A1 serpentine tube anode. J. Ind. Eng. Chem..

[11] Guidelines for Drinking Water Quality 4th ed. (World Health Organization (WHO), 2011).

[12] USEPA. National Primary Drinking Water Regulations: Consumer Factsheet on: Nitrate/nitrites (United States Environmental Protection Agency, 2010).

[13] WHO. Revisions of the WHO Guidelines for Drinking-Water Quality-Report on a WHO Consultation (2010).

[14] Smith VH, Tilman GD, Nekola JC (1999). Eutrophication: Impacts of excess nutrient inputs on freshwater, marine, and terrestrial ecosystems. Environ. Pollut..

[15] Quan X, Ye C, Xiong Y, Xiang J, Wang F (2010). Simultaneous removal of ammonia, P and COD from anaerobically digested piggery wastewater using an integrated process of chemical precipitation and air stripping. J. Hazard Mater..

[16] Raval HD, Rana PS, Maiti S (2015). A novel high-flux, thin-film composite reverse osmosis membrane modified by chitosan for advanced water treatment. RSC Adv..

[17] Yang HLG (2005). Chemical reduction of nitrate by nanosized iron: Kinetics and pathways. Water Res..

[18] Appunni S, Rajesh MP, Prabhakar S (2016). Nitrate decontamination through functionalized chitosan in brackish water. Carbohydr. Polym..

[19] Kuokkanen V, Kuokkanen T, Ramo J, Lassi U, Roininen J (2015). Removal of phosphate from wastewaters for further utilization using electrocoagulation with hybrid electrodes- techno-economic studies. J. Water Process Eng..

[20] Van Voorthuizen EM, Zwijnenburg A, Wessling M (2005). Nutrient removal by NF and RO membranes in a decentralized sanitation system. Water Res..

[21] Lin YF, Chen HW, Chen YC, Chiou CS (2013). Application of magnetic modified with polyacrylamide to adsorb phosphate in aqueous solution. J. Taiwan Inst. Chem. Eng..

[22] Song HO, Zhou Y, Li AM, Muller S (2012). Selective removal of nitrate from water by a microporous strong basic anion exchange resin. Desalination.

[23] Li QM, Fu LC, Wang Z, Li AM, Shuang CD, Gao CZ (2017). Synthesis and characterization of a novel magnetic cation exchange resin and its application for efficient removal of Cu^2+^ and Ni^2+^ from aqueous solutions. J. Clean Prod..

[24] Bergquist AM, Choe JK, Strathmann TJ, Werth CJ (2016). Evaluation of a hybrid ion exchange-catalyst treatment technology for nitrate removal from drinking water. Water Res..

[25] Martinez ME, Castillo JM, Yousfi EF (1999). Photoautotrophic consumption of phosphorus by *Scenedesmus obliquus*in a continuous culture. Influence of light intensity. Process Biochem..

[26] Karthikeyan P, Banu HAT, Preethi J, Meenakshi S (2020). Performance evaluation of biopolymeric hybrid membrane and their mechanistic approach for the remediation of phosphate and nitrate ions from water. Nature.

[27] Bassin JP, Kleerebezem R, Dezotti M, van Loosdrecht MCM (2012). Simultaneous nitrogen and phosphate removal in aerobic granular sludge reactors operated at different temperatures. Water Res..

[28] Wang H, Gao Q, Liu S, Chen Q (2020). Simultaneous nitrogen and carbon removal in a single biological aerated filter by the bioaugmentation with heterotrophic-aerobic nitrogen removal bacteria. Environ. Technol..

[29] Rossi F, Motta O, Matrella S, Proto A, Vigliotta G (2015). Nitrate removal from wastewater through biological denitrification with OGA 24 in a batch reactor. Water.

[30] Su JF, Zhang H, Hunag TL, Hu XF, Chen CL, Liu JR (2020). The performance and mechanism of simultaneous removal of fluoride, calcium and nitrate by calcium precipitating strain *Acinetobacter* sp. H12. Ecotoxicol. Environ. Saf..

[31] Li Y, Wang Y, Fu L, Goo Y, Zhao H, Zhou W (2017). Aerobic-heterotrophic nitrogen removal through nitrate reduction and ammonium assimilation by marine bacterium *Vibrio* sp. Y1–5. Bioresour. Technol..

[32] John EM, Krishnapriya K, Sankar TV (2020). Treatment of ammonia and nitrite in aquaculture wastewater by an assembled bacterial consortium. Aquaculture.

[33] Dalecka B, Strods M, Juhna T, Rajarao GK (2020). Removal of total phosphorus, ammonia nitrogen and organic carbon from non-sterile municipal wastewater with *Trametes versicolor* and *Aspergillus luchuensis*. Microbiol. Res..

[34] Hultberg M, Bodin H (2017). Fungi-based treatment of brewery wastewater- biomass production and nutrient reduction. Environ. Biotechnol..

[35] Robledo-Mahón T, Calvo C, Aranda E (2020). Enzymatic potential of bacteria and fungi isolates from the sewage sludge composting process. Appl. Sci..

[36] APHA. Standard methods for the examination of water and wastewater (American Public Health Association/American Water Works Association/Water Pollution Control Federation, 1998).

[37] Pokhriya P, Rajput R, Nautiyal P, Panwar P, Pandey D, Daverey A, Arunachalam A, Shridhar V, Arunachalam K (2020). Impact assessment of textile effluent on health and microbiota of agricultural soil in Bhagwanpur (Uttarakhand), India. SN Appl. Sci..

[38] Bhat BB, Mir RA (2015). Physiochemical assessment of soil in Doiwala Dehradun District of Uttarakhand (India). Int. J. Innov. Res. Dev..

[39] Watanable K (2001). Microorganisms relevant to bioremediation. Curr. Opin. Biotechnol..

[40] Campbell WA (1952). Methemoglobinemia due to nitrates in well-water. Br Med. J..

[41] Li H, Zhong Y, Huang H, Tan Z, Sun Y, Liu H (2020). Simultaneous nitrogen and phosphorus removal by interactions between phosphate accumulating organisms (PAO) and denitrifying phosphate accumulating organisms (DPAOs) in a sequencing batch reactor. Sci. Total Environ..

[42] Howarth RW, Marino R (2006). Nitrogen as the limiting nutrient for eutrophication in coastal marine ecosystems: Evolving views over three decades. Limnol. Oceanogr..

[43] Conley DJ, Paerl HW, Howarth RW, Boesch DF, Seitzinger SP, Havens KE, Lancelot C, Likens GE (2009). Controlling eutrophication: Nitrogen and phosphorus. Science.

[44] Dodds WK, Bouska WW, Eitzmann JL, Pilger TJ, Pitts KL, Riley AJ, Schloesser JT, Thornbrugh DJ (2009). Eutrophication of US freshwaters: Analysis of potential economic damages. Environ. Sci. Technol..

[45] Li H, Zhong Y, Huang H, Tan Z, Sun Y, Liu H (2020). Simultaneous nitrogen and phosphorus removal by interactions b/w phosphate accumulating organisms (PAOs) and denitrifying phosphate accumulating organisms (DPAOs) in a sequencing batch reactor. Sci. Total Environ..

[46] Ge Z, Wei D, Dhang J, Hu J, Liu Z, Li R (2018). Natural pyrite to enhance simultaneous long-term nitrogen and phosphorus removal in constructed wetland: Three years of pilot study. Water Res..

[47] Ye Y, Gan J, Hu B (2015). Screening of phosphorus-accumulating Fungi and their potential for phosphorus removal from waste streams. Appl. Biochem. Biotechnol..

[48] Posadas E, Encina PAG, Soltau A, Dominguez A, Diaz I, Munoz R (2013). Carbon and nutrient removal from centrates and domestic wastewater using algal-bacterial biofilm bioreactors. Bioresour. Technol..

[49] Kim J, Kang S, Kim HS, Kim S, Seoblee S (2020). Pilot Plant study on nitrogen and phosphorus removal in marine wastewater by marine sediment with sequencing batch rector. PLoS ONE.

[50] Chen C, Wang Z, Zhao M, Yuan B, Yao J, Chen J, Hrynshpan D (2021). A fungus-bacterium co-culture synergistically promoted nitrogen removal by enhancing enzyme activity and electron transfer. Sci. Total Environ..

[51] Papadopoulos KP, Economou CN, Dailianis S, Charalampous N, Stefanidou N, Gounic MM, Tekerlekopoulou AG, Vayenas DV (2020). Brewery wastewater treatment using cyanobacterial-bacterial settleable aggregates. Algal Res..

[52] He Q, Rajendran A, Gan J, Lin H, Felt CA, Hu B (2018). Phosphorus recovery from dairy manure wastewater by fungal biomass treatment. Water Environ. J..

[53] Bawiec A (2019). Efficiency of nitrogen and phosphorus compounds removal in hydroponic wastewater treatment plant. Environ. Technol..

[54] Lee CS, Lee SA, Ko SR, Oh HM, Ahn CY (2015). Effects of photoperiod on nutrient removal, biomass production and algal-bacterial population dynamics in lab-scale photobioreactors treating municipal wastewater. Water Res..

[55] Reza M, Cuenca MA (2016). Simultaneous biological removal of nitrogen and phosphorus in a vertical bioreactor. J. Environ. Chem. Eng..

[56] Hultberg M, Bodin H (2017). Fungi-based treatment of brewery wastewater-biomass production and nutrient reduction. Appl. Microbiol. Biotechnol..

[57] Liu S, Daigger GT, Liu B, Zhao W, Liu J (2020). Enhanced performance of simultaneous carbon, nitrogen and phosphorus removal from municipal wastewater in an anaerobic-aerobic-anoxic sequencing batch reactor (AOA-SBR) system by alternating the cycle times. Bioresour. Technol..

[58] Al-Dhabi NA, Esmail GA, Alzeer AF (2021). Removal of nitrogen from wastewater of date processing industries using a Saudi Arabian mesophilic bacterium, *Stenotrophomonas maltophilia* Al-Dhabi-17 in sequencing batch reactor. Chemosphere.

[59] Sniffen KD, Sales CM, Olson MS (2016). Nitrogen removal from raw landfill leachate by an algae-bacteria consortium. Water Sci. Technol..

[60] Zhang M, Pan L, Liu L, Su C, Dou L, Su Z, He Z (2020). Phosphorus and nitrogen removal by a novel phosphate-accumulating organism, *Arthrobacter* sp. HHEP5 capable of heterotrophic nitrification-aerobic denitrification: Safety assessment, removal characterization, mechanism exploration and wastewater treatment. Bioresour. Technol..

[61] Wang Y, Wang S, Sun L, Sun Z, Li D (2020). Screening of a *chlorella*-bacterium consortium and research on piggery wastewater purification. Algal Res..

[62] Liu Y, Zhu Y, Qiao X, Zheng B, Chang S, Fu Q (2018). Investigation of nitrogen and phosphorus contents in water in the tributaries of Danjiangkou reservoir. R. Soc. Open Sci..

[63] Coelho LM, Rezende HC, Coelho LM, de Sousa PAR, Melo DFO, Coelho NMM (2015). Bioremediation of polluted waters using microorganisms. Adv. Bioremediation Wastewaters Pollut. Soil..

[64] Zhang LH, Zeng GM, Dong HR, Chen YN, Zhang JC, Yan M, Zhu Y, Yuan YJ, Xie YK, Huang ZZ (2017). The impact of silver nanoparticles on the co-composting of sewage sludge and agricultural waste: Evolutions of organic matter and nitrogen. Bioresour. Technol..

[65] Jan V (2006). Removal of nutrients in various types of constructed wetlands. Sci. Total Environ..

[66] Rashida MI, Mujawar LH, Shahzad T, Almeelb T, Ismail IMI, Oves M (2016). Bacteria and fungi can contribute to nutrients bioavailability and aggregate formation in degraded soils. Microbiol. Res..

[67] McMahon KD, Dojka MA, Pace NR, Jenkins D, Keasling JD (2002). Polyphosphate Kinase from activated sludge performing enhanced biological phosphorus removal. Appl. Environ. Microbiol..

[68] Jiang X, Xin X, Li S, Zhou J, Zhu T, Muller C, Cai Z, Wright AL (2015). Effects of Fe oxide on N transformations in subtropical acid soils. Sci. Rep..

[69] Ahlgren J, Tranvik L, Gogoll A, Waldeback M, Markides K, Rydin E (2005). Sediment depth attenuation of biogenic phosphorus compounds measured by P-31 NMR. Environ. Sci. Technol..

[70] Reitzel K, Ahlgren J, Gogoll A, Rydin E (2006). Effects of aluminium treatment on phosphorus, carbon and nitrogen distribution in lake sediment: A P-31 NMR study. Water Res..

[71] Reitzel K, Ahlgren J, De Brabandere H, Waldeback M, Gogoll A, Tranvik L, Rydin E (2007). Degradation rates of organic phosphorus in lake sediment. Biogeochemistry.

[72] Tong S, Wang S, Zhao Y, Feng C, Xu B, Zhu M (2019). Enhanced alure-type biological system (E-ATBS) for carbon, nitrogen and phosphorus removal from slaughterhouse wastewater: A case study. Bioresour. Technol..

[73] Bhambhri A, Karn SK (2020). Biotechnique for nitrogen and phosphorus removal: A possible Insight. Chem. Ecol..

